# Biased Goiter Prevalence Estimate

**DOI:** 10.4103/0970-0218.55302

**Published:** 2009-07

**Authors:** MB Soudarssanane, P Punithakumary

**Affiliations:** Department of Preventive and Social Medicine, JIPMER, Pondicherry, India. E-mail: drmybase@gmail.com

Sir,

There are some basic queries related to the article ‘Prevalence of Goiter in Rural Area of Belgaum District, Karnataka’(1). In the introduction the authors should have made some mention of the magnitude of the problem in Karnataka from earlier studies or should have informed that no such study is available. Under materials and methods mention has been made as community based cross sectional study; a cross sectional study by nature is community based. In this study 950 subjects were contacted; 334 males and 616 females. The sex ratio is highly skewed and is not representative of any of the sex ratio across the country. The higher prevalence among females is represented in the higher overall prevalence which is a gross overestimate. Therefore, subsequent sub-analysis and multiple logistic regressions are unreliable. Though in the results 950 subjects are accounted for, yet a limitation is mentioned that those who were not available could not be examined which depicts serious uncertainty since the whole population of the two villages are not mentioned and the percentage unaccounted persons can not be assessed.
